# Rasch model analysis of the Depression, Anxiety and Stress Scales (DASS)

**DOI:** 10.1186/1471-244X-9-21

**Published:** 2009-05-09

**Authors:** Tracey L Shea, Alan Tennant, Julie F Pallant

**Affiliations:** 1 Faculty of Life and Social Sciences, Swinburne University of Technology, P.O. Box 218, Hawthorn, Victoria 3122, Australia; 2 Department of Rehabilitation Medicine, Faculty of Medicine and Health, University of Leeds, D Floor, Martin Wing, The General Infirmary at Leeds, Gt George Street, Leeds, LS1 3EX, UK; 3 School of Rural Health, University of Melbourne, 49 Graham Street, Shepparton Victoria 3630, Australia

## Abstract

**Background:**

There is a growing awareness of the need for easily administered, psychometrically sound screening tools to identify individuals with elevated levels of psychological distress. Although support has been found for the psychometric properties of the Depression, Anxiety and Stress Scales (DASS) using classical test theory approaches it has not been subjected to Rasch analysis. The aim of this study was to use Rasch analysis to assess the psychometric properties of the DASS-21 scales, using two different administration modes.

**Methods:**

The DASS-21 was administered to 420 participants with half the sample responding to a web-based version and the other half completing a traditional pencil-and-paper version. Conformity of DASS-21 scales to a Rasch partial credit model was assessed using the RUMM2020 software.

**Results:**

To achieve adequate model fit it was necessary to remove one item from each of the DASS-21 subscales. The reduced scales showed adequate internal consistency reliability, unidimensionality and freedom from differential item functioning for sex, age and mode of administration. Analysis of all DASS-21 items combined did not support its use as a measure of general psychological distress. A scale combining the anxiety and stress items showed satisfactory fit to the Rasch model after removal of three items.

**Conclusion:**

The results provide support for the measurement properties, internal consistency reliability, and unidimensionality of three slightly modified DASS-21 scales, across two different administration methods. The further use of Rasch analysis on the DASS-21 in larger and broader samples is recommended to confirm the findings of the current study.

## Background

According to the World Health Organisation (WHO) mental illness is prevalent, in all strata across all countries and societies [1]. Disorders such as schizophrenia, bipolar disorder, depression and anxiety and dementia related disorders are some of the main reasons individuals live with disability. Depression and anxiety are among the most common diagnoses in primary care and account for approximately 24% of diagnoses [2,3].

The importance of recognising and treating depression and anxiety cannot be understated as these conditions can result in a substantial reduction in perceived quality of life. This may manifest as restricted participation in the workplace, reduction in general health and dissatisfaction in family or social life [4-6]. Individuals with anxiety disorders are less likely to participate in the workforce compared to individuals with disabilities and long-term health problems [7], while those with depression are likely to be less productive at work or need to reduce the amount of work they perform [6,8]. Depression has been reported as the most important risk factor for suicide [9]. A study by Suominen, Henrikkson, Suokas et al [10] found that 38% of suicide attempters had been reported to have major depressive disorder while 75% were diagnosed with a depressive syndrome (e.g. major depression, depressive disorder not otherwise specified).

In addition to the personal burden associated with depression and anxiety, there are also substantial financial costs to the community [11,12]. Direct costs due to treatment are a major contributor to the economic burden of anxiety [11,13], however DuPont and colleagues [14] suggest that the greatest impact results from indirect costs such as lost productivity in the workplace.

The impact of untreated depression and anxiety on the ability to function is reported to be equal or greater than that of other common health problems such as heart disease or arthritis [15]. Timely and adequate treatment of these conditions is necessary as early detection may lead to better outcomes for the individuals concerned [16]. The importance of screening for depression and anxiety in younger populations has also been indicated, as early identification could potentially lead to a reduction in life-long mental health and social problems [17,18]. The routine use of screening instruments can substantially improve physician recognition of depression and anxiety disorders [15,19], increasing the likelihood of diagnosis and treatment [20].

### Measuring depression & anxiety

Several scales have been developed for the purpose of measuring depression and anxiety. These include the Beck Depression Inventory (BDI) [21], the Beck Anxiety Inventory (BAI) [22], the Hospital Anxiety and Depression Scale (HADS) [23], the Center for Epidemiological Studies Depression (CES-D) [24], and the Depression Anxiety Stress Scales (DASS) [25]. The most recent of these, the DASS, was originally developed for the purpose of measuring the distinctive aspects of depression and anxiety without either subscale being contaminated by the other construct. During the development phase a third subscale emerged that appeared to measure physiological stress. The result was a 42-item scale comprising three 14-items subscales that measure depression, anxiety and stress, a structure that was consistent with the tripartite model of anxiety and depression originally proposed by Watson and colleagues [26,27]. The DASS-21 was developed as a short form of the DASS-42 and has been reported to have slightly improved psychometric properties compared to the full DASS [28].

Subsequent studies have used factor analytic techniques to investigate the underlying structure of the DASS. The results from confirmatory factor analytic studies indicate that the original three factor structure rarely meets current standards regarding good model fit. The fit statistics reported by Lovibond and colleagues [25] fall below the minimally acceptable levels, while other studies, such as those by Henry and Crawford [29,30], achieved barely acceptable model fit only by allowing cross loadings between factors or correlating errors. The correlation of errors is a breach of local independence assumptions. This requires that the indicators of a latent construct are independent given the (correctly specified) latent variable model [31]. While correlating errors appears to be common practice within a CFA framework, and might be theoretically justified in some instances, the validity of this practice has been challenged [32]. Duffy, et al [33] concluded that the DASS may be better represented by a two factor structure which they termed general negativity and physiological arousal. Alternatively, Henry and Crawford [30] suggest that the DASS-21 may be best represented by one common underlying factor, which they described as 'general psychological distress'.

The psychometric evaluation of the DASS to date has been conducted within the framework of classical test theory. Over the last ten years however there has been a growing awareness in the health and psychological sciences of modern test theory approaches, such as those based on the Rasch measurement model. Rasch analysis allows a detailed investigation of many aspects of a scale including the response format, the fit of individual items and persons, dimensionality, targeting, and the detection of item bias. Testing for differential item functioning is particularly important as it allows researchers and clinicians to ensure that items function uniformly across age, gender or, for example, different scale administration methods, at all difficulty levels.

### Method of administration

The internet has been used to raise awareness of mental illness in the general public by providing self-administered tests online. As a result, scales such as Web-Based Depression and Anxiety Test [34] and the Center for Epidemiological Studies Depression scales [24] have been developed or adapted for internet use. However, it has been argued that the administration of online scales might differ from pencil-and-paper administration in both presentation and in the way in which questions are answered [35]. How this may affect item functioning is unclear and further investigation is required when converting a pencil-and-paper measure to an online version.

Research investigating the use of web surveys across a variety of disciplines has suggested that this method of data collection can be cost effective, decreases turn-around time for data collection [36] and provides researchers with the ability to download data electronically [37]. McCabe and colleagues [38,39] also reported increased response rates and no difference in prevalence rates for paper-and-pencil versus web-based surveys.

Despite these advantages, problems such as missing data, differences in mean scores between web-based and pencil and paper surveys and a potentially biased sample due to the need for internet access [40] have been reported. Some studies investigating the impact of scale administration methods have reported similar psychometric properties for pencil-and-paper surveys and their web-based counterparts [41-43]. However, sampling from the web may restrict the types of persons who respond to web-based surveys, as these respondents may not be socially or economically representative of the general population [44]. Although the adaptation of scales for web delivery appears promising, it is important to ensure that the new web-based version of a scale does in fact display equivalent psychometric properties to its pencil-and-paper counterpart.

In addition to the practical psychometric issues associated with online surveys, another concern raised in the literature is whether participant response styles vary between administration methods [35]. Some authors have suggested that respondents participating in web-based surveys might be more disinhibited in their responses compared to those who complete pencil-and-paper surveys [45]. This indicates a need, not just for good screening tools, but also a sound understanding of the impact of their associated methodologies.

Only one study thus far has used a web-based data collection method for the DASS-21 [46]. Wong and colleagues [46] reported that a web-based design was well received by their participant sample and did not have a negative impact on their response rate. However, this study did not have a pencil-and-paper comparison group, therefore it is not known how the web-based version of the DASS-21 performed in comparison to a more traditional method of data collection.

### Aims and objectives

While there have been a number of studies finding support for the psychometric properties of the DASS-21 no studies to date have used Rasch analysis. The first aim of this study was therefore to apply Rasch analysis to the three DASS-21 subscales to conduct a detailed assessment of the response format, item fit, dimensionality and targeting. The suitability of using all items of the DASS-21 as a measure of general psychological distress was also explored. Finally, Rasch analysis was used to determine whether the web-based version of the DASS-21, compared to a pencil-and-paper version, would introduce item bias due to the use of an alternative administration technique.

## Methods

### Participants

Respondents were recruited to complete either a pen-and-paper version or a web-based version of the DASS-21 in a large stress resilience study of 745 participants. The web-based respondents were recruited from a variety of organizations (including employees of schools, hospitals, small businesses) in Melbourne, Australia. The pen-and-paper sample was obtained by inviting the staff, students (and associated family members) of Swinburne University, Melbourne, to complete a questionnaire booklet. In this study 210 respondents completed a pencil and paper questionnaire and 535 respondents completed a web-based questionnaire. The present study consists of the 210 respondents who completed the pen and paper version of the DASS-21 and a random selection of 210 respondents from the original 535 respondents who completed the web-based version. This was done to ensure an equal number of respondents who had completed the pen-and-paper and web-based versions of the DASS-21 to comply with the requirements of the ANOVA based analysis for invariance of the items across groups. The questionnaires were anonymous and no financial remuneration was offered to participants. The study was approved by the Swinburne University Ethics Committee.

### Measures

The short version of the DASS (DASS-21) was used in this study [25]. It consists of three seven-item scales measuring depression, anxiety and stress. Participants were asked to read each statement carefully and to indicate how much each statement applied to them over the past week. The response categories for each scale ranged from 0 to 3 (0 = did not apply to me at all; 1 = applied to me to some degree, or some of the time; 2 = applied to me to a considerable degree, or a good part of time; and 3 = applied to me very much, or most of the time). Responses to each scale item were summed to produce a total score for that scale.

This scale was developed by Lovibond and colleagues [25] and has undergone extensive evaluation by the authors and other research groups [28,29,33,47]. Analysis of the DASS-21 has consistently presented a three factor structure as the optimal solution. The items of the depression scale focus on low mood, low self-esteem and poor outlook for the future. The anxiety scale items focus on a fear response and physiological arousal while the stress subscale focuses on persistent arousal and tension. The DASS-21 used in this study can be downloaded from: 

### Statistical analysis

Rasch analysis in this study was conducted using RUMM 2020 [48]. The DASS-21 was analysed in two stages. The first stage was to subject the individual DASS-21 scales to Rasch analysis. Following this, Rasch analysis was performed on all items of the DASS-21 to evaluate the validity of using a total score from this scale as a measure of general psychological distress.

The process of Rasch analysis is described in detail elsewhere [49,50]. Briefly the task is to test if the observed pattern of responses to items conforms to the Rasch model expectations, as the model defines how such responses should be if interval scale data is to be constructed [51]. Consequently the analysis is concerned with tests of fit, and tests of assumptions such as unidimensionality. Where these tests are satisfied, and the assumptions hold, the scale can be viewed as a unidimensional Rasch scale, and the raw score (obtained by summing the items) can be transformed into interval scaling.

Initially, because the DASS-21 has polytomous response options, a likelihood ratio test was conducted for each subscale to determine whether it was more appropriate to use the Rating Scale version of the model [52] or the Partial Credit version [53]. In the former, the expectation is that within a set of polytomous items, the response categories are defined *and *function in the same way for each item [54]. Consequently, in the latter the response categories may vary in both definition and/or function across items. By function, it is meant that the distances between the transition points across categories (thresholds – signifying the point between adjacent response categories where either response is equally probable) are the same across all items. The likelihood ratio test determines whether this is the case, and so determines which model is appropriate. The suitability of the response format itself was assessed by inspecting the item thresholds. All items are expected to have ordered response thresholds, thus consecutive thresholds are expected to demonstrate an increase along the underlying trait. Where this does not happen, thresholds are said to be disordered, and this is usually resolved by combining response categories [55].

Several tests of fit were used in the current study including overall summary tests of fit, as well as individual item and person tests. The main aim of these tests was to show that the responses do not deviate from Rasch model expectations. Thus a summary chi-square interaction fit statistic should be non significant, as should the individual item chi-square statistics after Bonferroni adjustment to the alpha level. This adjustment is required because multiple tests are performed, one for each chi-square statistic for each item [56]. Consequently some items may be shown to misfit model expectations just by chance, particularly when the number of items, and thus tests undertaken, is large.

The standard deviation of the summary residual statistic should also not deviate too much from 1 (perfect fit), and certainly should not be above 1.5. Individual item residuals should fall within the range ± 2.5 (99% confidence level). High positive fit residual values indicate misfit, while high negative fit residuals suggest item redundancy. Items were also examined for Differential Item Functioning (DIF) across subgroups within the sample (age, gender, education and scale administration method) using analysis of variance with a Bonferroni adjusted alpha level.

The three DASS-21 scales, and the DASS-21 as a measure of general psychological distress, were evaluated to determine how well targeted the items were for the sample and to assess whether the individual scales and the DASS-21 as a whole represented unidimensional constructs. Unidimensionality was tested using the approach suggested by Smith [57]. Person estimates, derived from subsets of items identified by high positive and negative loadings on the first principal component of the residuals, were tested for significant differences. Using a series of independent t-tests, if more than 5% of these tests are significant (or specifically the lower bound of the binomial confidence interval is above 5%), the scale is deemed to be multidimensional. This approach has been shown to be robust to simulated levels of multidimensionality in polytomous scales [58]. A Person Separation Index (PSI) value was calculated for each scale, with values of .7 or above indicating adequate internal consistency.

## Results

The sample consisted of a total of 420 respondents with 210 (50%) completing the pencil and paper version of the DASS-21 and 210 (50%) completing the web-based version of the scale. In each group approximately one third of the sample were males (see Table 1). Respondents were classified into three age groups for DIF analysis (29 years or younger, 30–40 years, and 41 years and older). Respondent's level of education was recorded as non-tertiary, undergraduate or postgraduate.

**Table 1:** Participant demographics

	**Pencil & Paper** **(n = 210)**	**Web-based** **(n = 210)**
*Gender*		
Male	80 (38%)	62 (30%)
Female	130 (62%)	148 (70%)
*Age*		
Less than 30 years	83 (40%)	81 (39%)
30 – 40 years	45 (21%)	67 (32%)
More than 40 years	81 (39%)	62 (30%)
*Education*		
Non-tertiary	101 (48%)	65 (31%)
Undergraduate	73 (35%)	59 (28%)
Postgraduate	34 (16%)	86 (41%)

For the Rasch analysis of each subscale, the likelihood ratio tests were significant indicating that it was more appropriate to use the Partial Credit version of the Rasch model.

### DASS-21-Depression

Initial analysis of the seven item depression scale revealed poor model fit (p < .001) (see Table 2 – Analysis 1). No serious misfit was observed for persons, but the mean fit residual value for items (2.6) suggested the presence of misfitting items. No disordered thresholds were observed, providing support for the response format. Individual item fit scores revealed two items with significant chi-square probability values (items 5 and 10), while item five also had a very high positive fit residual value (4.76) suggesting misfit. Deleting item five (*I found it difficult to work up the initiative to do things*) improved model fit (p = .03) (Table 2 – Analysis 2), with no misfitting items (Table 3). The PSI value of .89 indicated good person reliability. No evidence of differential item functioning was observed for age, education or scale administration method. Differential item functioning by gender was evident for items 13 and 16. At equivalent levels of depression, female respondents endorsed item 13 at a higher level than male respondents. Conversely, at equivalent levels of depression male respondents endorsed item 16 at a higher level compared to female respondents. No further action was taken as the level of DIF was relatively minor and likely to cancel out at the subscale level. There was no evidence of multidimensionality with a series of independent t-tests, comparing person estimates from subtests identified using PCA of the residuals, indicating only 1.45% statistically significant tests. With a mean depression location of -2.34 (SD 1.79) the scale is off-target for this sample as the majority are showing little or no depressive symptoms, with 35% at the floor of the scale.

**Table 2:** Model fit statistics for original and revised DASS-21 scales

**Action**	**Analysis**	**Overall** **model fit**	**Item Fit Resid Mean (SD)**	**Person Fit Resid Mean (SD)**	**PSI**	**% signif t-tests**
**DASS-21-Depression**						
1. Original scale	1	χ^2 ^= 86.68 p < .001	-0.73 (2.60)	-0.34 (0.90)	0.87	
2. Removal of item 5	2	χ^2 ^= 38.41 p = .03	-0.21 (0.84)	-0.34 (0.93)	0.89	1.45%
**DASS-21-Anxiety**						
3. Original scale	3	χ^2 ^= 69.76 p < .001	-0.69 (1.51)	-0.34 (0.95)	0.76	
4. Removal of item 2	4	χ^2 ^= 40.57 p = .02	-0.52 (0.89)	-0.34 (1.12)	0.77	2.77%
**DASS-21-Stress**						
5. Original scale	5	χ^2 ^= 57.47 p < .001	-0.29 (1.69)	-0.40 (1.30)	0.84	
6. Removal of item 11	6	χ^2 ^= 42.09 p = .01	-0.27 (1.36)	-0.40 (1.22)	0.80	3.36%
**DASS-21-Total**						
7. Original scale	7	χ^2 ^= 236.4 p < .001	-0.46 (2.33)	-0.30 (1.27)	0.90	
8. Removal of items 1,2,5,19,14	8	χ^2 ^= 100.19 p = .003	-0.46 (1.49)	-0.32 (1.21)	0.90	11.61% CI:9–14%
**DASS-21-Anxiety/Stress**						
9. Original scale	9	χ^2 ^= 159.20 p < .001	-0.47 (2.18)	-0.31 (1.15)	0.87	
10. Removal of items 2, 11, 15	10	χ^2 ^= 60.90 p = .046	-0.55 (1.27)	-0.36 (1.08)	0.84	3.07%

SE = Standard error, Fit Resid = Fit Residual, ChiSq = Chi-square, p = probability, PSI = Person Separation Index

**Table 3:** Individual item fit statistics for the final models of the revised DASS-21 subscales

**Item no.**	**Item**	**Location value**	**SE**	**Fit Resid**	**ChiSq**	**Prob**
	**DASS-21-Depression**					
3	I couldn't seem to experience any positive feeling at all	0.059	0.101	0.742	3.107	0.54
10	I felt that I had nothing to look forward to	0.048	0.097	-1.477	8.234	0.08
13	I felt down-hearted and blue	-1.100	0.089	0.685	5.280	0.26
16	I was unable to become enthusiastic about anything	-0.120	0.097	-0.284	8.884	0.06
17	I felt I wasn't worth much as a person	0.296	0.099	-0.248	3.719	0.45
21	I felt that life was meaningless	0.817	0.109	-0.680	9.191	0.06
	**DASS-21-Anxiety**					
4	I experienced breathing difficulty	0.115	0.086	-0.210	7.169	0.13
7	I experienced trembling (e.g. in the hands)	0.108	0.086	-0.413	5.858	0.21
9	I was worried about situations in which I might panic and make a fool of myself	-0.483	0.077	0.361	2.220	0.70
15	I felt I was close to panic	0.061	0.087	-1.836	13.075	0.01
19	I was aware of the action of my heart in the absence of physical exertion	-0.100	0.083	0.304	5.257	0.26
20	I felt scared without any good reason	0.298	0.090	-1.311	6.987	0.14
	**DASS-21-Stress**					
1	I found it hard to wind down	-0.552	0.070	0.600	6.544	0.16
6	I tended to over-react to situations	0.001	0.070	0.313	3.490	0.48
8	I felt that I was using a lot of nervous energy	0.313	0.070	0.107	3.117	0.54
12	I found it difficult to relax	-0.145	0.072	-2.648	14.029	0.007
14	I was intolerant of anything that kept me from getting on with what I was doing	0.346	0.074	1.059	4.195	0.38
18	I felt that I was rather touchy	0.038	0.072	-1.033	10.712	0.03

SE = Standard error, Fit Resid = Fit Residual, ChiSq = Chi-square, prob = probability

Anxiety: Fit Residual df = 208, Chi square df = 4

Depression: Fit Residual df = 226.33, Chi square df = 4

Stress: Fit Residual df = 319.67, Chi square df = 4

### DASS-21-Anxiety

Rasch analysis of the anxiety scale revealed poor model fit (Table 2 – Analysis 3). Items four (*I experienced breathing difficulty*) and seven (*I experienced trembling e.g. in the hands*) displayed disordered thresholds however rescoring of these items did not improve model fit so original scoring was retained. Three items (items 2, 15 and 20) had significant chi-square probability values and item 15 (*I felt I was close to panic*) had an extreme negative fit residual value (-2.57). The deletion of item 2 (*I was aware of dryness of my mouth*) resulted in an improved overall model fit (p = .02) and item fit residuals (Table 2 – Analysis 4), with no misfitting items (Table 3). There was no evidence of differential item functioning for the demographic variables (age, gender and education) or the scale administration method. The final PSI was 0.77, suggesting sufficient person separation reliability for group use only. Independent t-tests, comparing person estimates from subtests identified using PCA of the residuals, resulted in only 2.77% statistically significant tests, supporting the unidimensionality of the scale. In this sample the scale was off-target with a mean location on the latent trait of anxiety of -1.78 (SD 1.01), and with 40% displaying no symptoms of anxiety.

### DASS-21-Stress

Analysis of the stress scale items showed misfit to the Rasch model (p < .001) (Table 2 – Analysis 5). No items had significant chi-square values (after Bonferroni adjustment) or extreme positive fit residuals, however, two items (items 11 and 12) had high negative fit residuals (-2.47 and -2.49 respectively) indicating that these items overfit the model. The best solution was obtained by the deletion of item 11 (*I found myself getting agitated*) (p = .01). Although item 12 recorded a significant individual chi square value (p = .007) removal of this item resulted in a decrease in overall model fit (p = .002), therefore it was retained (Table 2 – Analysis 6). The final PSI was 0.80, suggesting reasonable person separation reliability. There was no evidence of differential item functioning for age, gender, education or scale administration method. There was support for the unidimensionality of the scale with independent t-tests, comparing person estimates from subtests identified using PCA of the residuals, indicating only 3.36% of cases showing statistically significant differences. The scale was slightly off target with a mean estimate on the latent variable of stress at -1.03 (SD = 1.35), with just 8% of the sample at the floor of the scale.

### DASS-21 – Total

All 21 items of the DASS-21 were subjected to Rasch analysis and overall fit statistics suggested substantial misfit to the model (p < .001) (Table 2 – Analysis 7). Person fit statistics were within an acceptable range but the item fit statistics indicated the presence of misfitting items. Disordered thresholds were observed for five items (items 4, 7, 17, 20 and 21) however, rescoring did not result in improved model fit therefore the original scoring was retained. In order to improve model fit five items were deleted (items: 2, 1, 5, 19, 14). The final PSI value was .90 indicating good person reliability separation (Table 2 – Analysis 8). PCA of the residuals showed strong positive loadings of the depression items on the first factor extracted, with stress and anxiety items loading negatively on the second factor. A series of independent t-tests on the person estimates derived from the positively and negatively loading items revealed 11.61% (CI: 9–14%) of cases with statistically significant t-values. This indicated the presence of multidimensionality, with the depression items tapping a different underlying trait than the remaining items.

**Figure 1:**
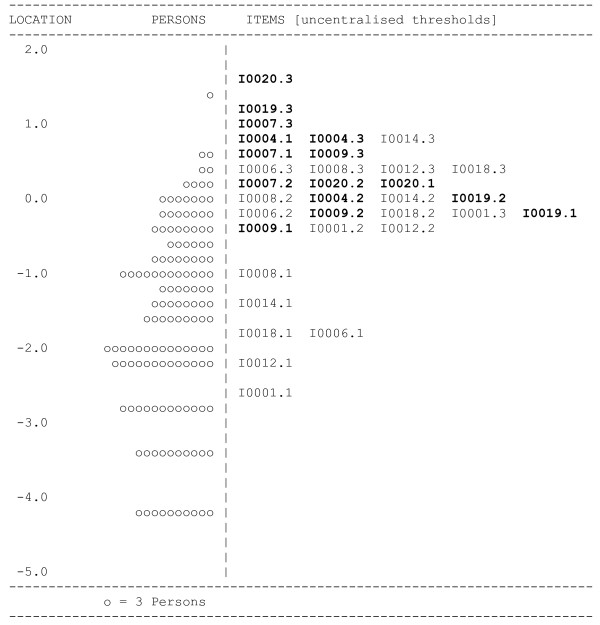
**Item map of the revised DASS Anxiety/Stress Subscale**. Note: Items from the Anxiety subscale are in bold font; items from the Stress subscale are in normal font.

Given the results of the PCA analysis, it was decided to assess the ability of the anxiety and stress items to form a unidimensional scale, separate from the depression items. Initial analysis revealed poor model fit (p < .001) (Table 2 – Analysis 9). After removal of three items (items 2, 11, 15) satisfactory fit to the Rasch model was achieved (p = .05) with a good person separation reliability of 0.84 (Table 2 – Analysis 10), and no misfitting items (see Table 4). No differential item functioning for age, gender, education or scale administration method was detected. There was no evidence of multidimensionality with independent t-tests, comparing person estimates from subtests identified using PCA of the residuals, showing only 3.07% of cases with significant differences. The item map in Figure 1 shows a separation of the anxiety and stress in terms of their relative difficulty. Anxiety items tend to be more difficult to endorse, appearing towards the top of the display, while the easiest items shown at the bottom are the stress items. The scale was off target for this sample with a mean estimate on the latent variable of stress of -1.46 (SD = 1.28).

**Table 4:** Individual item fit statistics for the DASS Anxiety/Stress Subscale (final model)

**No.**	**Item**	**Scale**	**Location value**	**SE**	**Fit Resid**	**ChiSq**	**Prob**
1	I found it hard to wind down	S	-0.960	0.068	1.195	3.738	0.443
4	I experienced breathing difficulty	A	0.636	0.082	0.289	2.077	0.722
6	I tended to over-react to situations	S	-0.439	0.069	-0.138	3.421	0.490
7	I experienced trembling (e.g. in the hands)	A	0.633	0.082	-1.310	3.083	0.544
8	I felt that I was using a lot of nervous energy	S	-0.133	0.069	-2.026	8.704	0.069
9	I was worried about situations in which I might panic and make a fool of myself	A	0.105	0.070	-0.134	2.664	0.616
12	I found it difficult to relax	S	-0.603	0.070	-2.686	10.091	0.039
14	I was intolerant of anything that kept me from getting on with what I was doing	S	-0.092	0.073	1.011	9.837	0.043
18	I felt that I was rather touchy	S	-0.419	0.070	-0.835	7.606	0.107
19	I was aware of the action of my heart in the absence of physical exertion	A	0.471	0.077	0.310	4.659	0.324
20	I felt scared without any good reason	A	0.802	0.085	-1.759	5.025	0.285

SE = Standard error, Fit Resid = Fit Residual, ChiSq = Chi-square, prob = probability

Scale: S = Stress scale item, A = Anxiety scale item

Fit Residual df = 352.55, Chi square df = 4

## Discussion

Depression screening instruments are now widely used in both clinical practice and research. Increasingly the scales have been scrutinized by a mix of classical and modern psychometric approaches. The purpose of the present study was to use Rasch analysis to assess the psychometric properties of the DASS-21 scales, and specifically to evaluate two different administration methods (pen and paper test, internet delivery). The possibility that the combined items of the DASS-21 could represent a valid measure of general psychological distress was also investigated.

Initially none of the three DASS-21 scales satisfied the criteria for fit to the Rasch model. To achieve satisfactory fit it was necessary to remove item 5 (*I found it difficult to work up the initiative to do things*) from the Depression scale, item 2 (*I was aware of dryness of my mouth*) from the Anxiety scale and item 11 (*I found myself getting agitated*) from the Stress scale. All revised scales showed adequate internal consistency, and no evidence of multidimensionality. There was no differential item functioning by administration method for any of the scales showing that the items are invariant across mode of administration, and therefore comparable.

Earlier studies, using CFA, have rarely deleted items to achieve model fit, they have instead performed statistical manipulations (e.g. correlated errors [30]) or presented alternative models [33,59]. Therefore, comparisons to earlier studies of the DASS-21 regarding problematic items are by necessity somewhat limited. Antony and colleagues [28] did not eliminate scale items. However, two of the items that were removed in the present study (item two and item five) were observed to have low factor loadings in that study [28] suggesting that they were not strongly correlated with their underlying constructs. Clara and colleagues [59] reported poor model fit for the original version of the DASS-21 but provided no specific details regarding misfitting items. Their final model was comprised of the original DASS-21 items and others from the full DASS-42 and is therefore not directly comparable to this study.

Removing items from well established scales is contentious, particularly those which have established clinical cut points and thus where this would change with a reduced item set [49]. This may be less problematic for new scales, where psychometric evidence is still accumulating. The Rasch model is very strict in that it is assessing the requirements for a transformation to interval scaling. Consequently a scale could fail this requirement, but still be a valid unidimensional ordinal scale. Given a response to all items, then cut points would be valid, as they only require a magnitude of the trait, which is consistent with ordinal scaling. However, given fit to the Rasch model, this cut point is then placed upon a metric and becomes independent of the set of items taken, and is thus directly transferable to any such set of items calibrated on the same metric.

Reducing existing item sets, as in the case of the reduction of the DASS-42 to the DASS-21 is an important issue. Modern psychometric approaches may give a different view of item reduction, and thus different short-forms may emerge as a result. This has the potential for causing confusion for users. Unfortunately, where short forms are shown to fail modern psychometric standards this becomes problematic and decisions may have to be taken to reduce the item set further (as we have done here reducing each DASS-21 scale by one item) or hope that the misfit to the model will not bias results of the original scale in any meaningful way. Given just one item was removed from each subscale, it would seem that at the present time the existing structure of the scale, while not ideal, may suffice in an ordinal scale format until such a time that further evidence accrues to suggest a revision of the item content. Where a transformation to interval scaling is required, then the current solution derived from the Rasch model is most appropriate. Another option would be to revisit the original full scale (DASS-42) and see if another short set of items would better satisfy modern standards.

The proposition that the combined items of the DASS-21 could be used as a measure of general psychological distress [30] was not supported in the current study. Multidimensionality was clearly evident with the depression items forming one subscale and the anxiety and stress items forming a second subscale. When person estimates generated from these two sets of items were compared over 11% of people recorded statistically significant differences in their scores. These results suggest that it is not appropriate to use the total scale as a single measure of general psychological distress.

Although it was not found to be appropriate to combine all three subscales, additional analysis in the current study suggested that the anxiety and stress subscales could form an anxiety-stress continuum. While it was necessary to remove three items from this combined scale, the model showed adequate fit, good internal consistency and no evidence of multidimensionality. This proposal is not consistent with the structure proposed by Duffy and colleagues [33] who suggested a two factor structure with one subscale comprised largely of depression and stress items and a second subscale comprised of anxiety items. The results of the present analysis would rather suggest that the anxiety and stress items lay along a single continuum, with stress at the lower end, and anxiety at the higher end. It could be argued that the anxiety items have a greater conceptual similarity to the stress items rather than the depression items. Further exploration of this proposal is needed on larger, and broader samples.

There are limitations to the current study. The targeting of the sample was less than desirable, in that significant floor effects were observed for the anxiety and depression subscales. Although this may simply reflect the distribution of symptoms in the general population, and is not a problem for a screening instrument, it can affect the precision of the item estimates, particularly items representing severe levels of anxiety and depression, where relatively few respondents were found. However, Linacre [60] has shown that a sample size of 243 is sufficient to give a degree of precision of ± 0.5 logits, at 99% confidence, even when poorly targeted. The sample sizes in this study, even after excluding extremes, were sufficient for this degree of precision. Nevertheless, replication in better targeted samples, with higher levels of anxiety and depression, would further support the robustness of the current findings.

## Conclusion

This was the first study to undertake a rigorous examination of the psychometric properties of the DASS-21 using Rasch analysis and to assess item bias by mode of administration (pen and pencil versus web-based). The results provide support for the measurement properties, internal consistency reliability, targeting, and unidimensionality of the three DASS-21 scales. However it was necessary to remove one item from each of the scales to achieve fit to the Rasch model. No differential item functioning was found for sex, age, education or mode of administration. The summation of all items to form a total scale representing general psychological distress was not supported, however a scale combining anxiety and stress items showed adequate psychometric properties. Further examination of fit of data from the DASS-21 to the Rasch measurement model in larger and appropriately targeted samples is recommended to confirm the findings of the current study.

## Abbreviations

DASS: Depression, Anxiety and Stress Scales; DIF: Differential Item Functioning; EFA: Exploratory Factor Analysis; PCA: Principal Components Analysis; PSI: Person Separation Index.

## Competing interests

The authors declare that they have no competing interests.

## Authors' contributions

TS conducted the literature review, performed the data analysis and prepared the first draft of the manuscript. AT participated in the data analysis and preparation of the manuscript. JP designed the study, collected the data, and supervised the data analysis and preparation of the manuscript. All authors read and approved the final manuscript.

## Pre-publication history

The pre-publication history for this paper can be accessed here:


